# Proteomic expression profiling of *Haemophilus influenzae *grown in pooled human sputum from adults with chronic obstructive pulmonary disease reveal antioxidant and stress responses

**DOI:** 10.1186/1471-2180-10-162

**Published:** 2010-06-01

**Authors:** Jun Qu, Alan J Lesse, Aimee L Brauer, Jin Cao, Steven R Gill, Timothy F Murphy

**Affiliations:** 1 Department of Pharmaceutical Sciences, School of Pharmacy and Pharmaceutical Sciences, University at Buffalo, State University of New York, Buffalo, NY 14260 USA; 2 Division of Infectious Diseases, Department of Medicine, School of Medicine and Biomedical Sciences, University at Buffalo, State University of New York, Buffalo, NY 14215 USA; 3 Department of Microbiology, School of Medicine and Biomedical Sciences, University at Buffalo, State University of New York, Buffalo, NY 14215 USA; 4 Department of Pharmacology & Toxicology, School of Medicine and Biomedical Sciences, University at Buffalo, State University of New York, Buffalo, NY 14215 USA; 5 Department of Oral Biology, School of Dental Medicine, University at Buffalo, State University of New York, Buffalo, NY 14215 USA; 6 New York State Center of Excellence in Bioinformatics and Life Sciences, 701 Ellicott Street, Buffalo NY 14203 USA; 7 Veterans Affairs Western New York Healthcare System, 3495 Bailey Avenue, Buffalo, New York 14215 USA

## Abstract

**Background:**

Nontypeable *Haemophilus influenzae *colonizes and infects the airways of adults with chronic obstructive pulmonary disease, the fourth most common cause of death worldwide.Thus, *H. influenzae*, an exclusively human pathogen, has adapted to survive in the hostile environment of the human airways.To characterize proteins expressed by *H. influenzae *in the airways, a prototype strain was grown in pooled human sputum to simulate conditions in the human respiratory tract.The proteins from whole bacterial cell lysates were solubilized with a strong buffer and then quantitatively cleaned with an optimized precipitation/on-pellet enzymatic digestion procedure.Proteomic profiling was accomplished by Nano-flow liquid chromatography/mass spectroscopy with low void volume and high separation efficiency with a shallow, long gradient.

**Results:**

A total of 1402 proteins were identified with high confidence, including 170 proteins that were encoded by genes that are annotated as conserved hypothetical proteins.Thirty-one proteins were present in greater abundance in sputum-grown conditions at a ratio of > 1.5 compared to chemically defined media.These included 8 anti-oxidant and 5 stress-related proteins, suggesting that expression of antioxidant activity and stress responses is important for survival in the airways.Four proteins involved in uptake of divalent anions and 9 proteins that function in uptake of various molecules were present in greater abundance in sputum-grown conditions.

**Conclusions:**

Proteomic expression profiling of *H. influenzae *grown in pooled human sputum revealed increased expression of antioxidant, stress-response proteins and cofactor and nutrient uptake systems compared to media grown cells.These observations suggest that *H. influenzae *adapts to the oxidative and nutritionally limited conditions of the airways in adults with chronic obstructive pulmonary disease by increasing expression of molecules necessary for survival in these conditions.

## Background

Nontypeable *Haemophilus influenzae *is an exclusively human pathogen whose primary ecological niche is the human respiratory tract.*H. influenzae *causes lower respiratory tract infections, called exacerbations, in adults with chronic obstructive pulmonary disease (COPD) and these infections cause substantial morbidity and mortality [1].In addition to causing intermittent acute infections in the setting of COPD, *H. influenzae *also chronically colonizes the lower airways in a subset of adults with COPD [2-4].In the normal human respiratory tract, the airways are sterile below the vocal cords.However, in adults with COPD the lower airways are colonized by bacteria, with *H. influenzae *as the most common pathogen isolated in this setting.This chronic colonization contributes to airway inflammation that is a hallmark of COPD [5,6].Thus, *H. influenzae *appears to be uniquely adapted to survive in the human respiratory tract of adults with COPD.

The human respiratory tract is a hostile environment for bacteria.Nutrients and energy sources are limited and the human airways express myriad antimicrobial peptides and molecules that are highly bactericidal [7-9]. Furthermore, the airways in adults with COPD are characterized by an oxidant/antioxidant imbalance which is an important component of the airway inflammation that characterizes COPD [10,11]. Thus, to survive and grow in the respiratory tract, bacteria must use energy sources and nutrients that are available and synthesize necessary metabolites.In addition, bacteria must express proteins and other molecules to enable persistence in spite of oxidative and inflammatory conditions and various antimicrobial substances that are active in the airways.Little is known about the mechanisms by which *H. influenzae *survives and multiplies in the human respiratory tract.

The goal of the present study is to characterize the proteome of *H. influenzae *during growth in pooled human sputum in an effort to partially simulate conditions that are present in the human respiratory tract.COPD is a disease entity that includes chronic bronchitis and emphysema.The major criterion that defines chronic bronchitis is chronic sputum production due to excess mucus production in the airways that results from hypertrophy of submucosal glands.Thus, the approach that we have taken is to grow a prototype COPD clinical isolate of *H. influenzae *in a chemically defined medium to which pooled sputum from adults with COPD has been added.The proteome of sputum-grown *H. influenzae *was characterized and compared to that of *H. influenzae *grown in chemically defined medium alone.Identifying proteins that demonstrate increased expression during growth in pooled human sputum will help to identify potential virulence factors or abundantly expressed surface antigens that, with further study, could lead to an understanding of the mechanisms by which *H. influenzae *survives and causes infection in the human respiratory tract.Understanding these mechanisms and elucidating the molecules that are expressed abundantly by *H. influenzae *when it grows in the respiratory tract may lead to the development of novel strategies for treatment or prevention of respiratory tract infections caused by *H. influenzae*.

The approaches generally employed for comparing proteomes include two-dimensional (2D) gel electrophoresis [12] and LC/MS-based methods, such as isotope labeling by metabolic incorporation (e.g. SILAC) [13,14] and chemical/enzymatic labeling(e.g. ICAT, iTRAQ and ^18^O-incorporation) [15-17], and more recently, label-free protein expression profiling approaches [18-24].Label-free methods employ a "shotgun" approach that is particularly effective for large-scale protein analysis [25] and carries the potential for providing higher quantitative accuracy (as demonstrated by the Association of Biomolecular Resource Facilities, http://www.abrf.org/prg). In addition, the label-free approach enables the ability to quantify and compare multiple biological/technical replicates, as required in this work. Therefore, in this study we employed the label-free expression profiling strategy we developed [26-29] for the relative quantification of proteins expressed at the two different culture conditions.

## Results and Discussion

### Expression profiling method optimization and evaluation

Because the label-free proteomic analysis approach often does not employ internal standards, quantitative and reproducible sample preparation, as well as robust, comprehensive and reproducible LC/MS analysis is particularly important for obtaining reliable results [30].To approach the difficulties associated with efficient protein extraction and sample cleanup, comprehensive protein identification, and reproducible quantification, we developed, optimized and evaluated the expression profiling procedure [29,31].

### Treatment of the bacterial samples

For label-free expression profiling of bacterial samples, an efficient and quantitative extraction of proteins from the biological matrix is critical. Therefore, a strong buffer that contains relatively high concentrations of both ionic and non-ionic detergents was employed (See Methods).Because most of the buffer components are not compatible with the subsequent digestion and LC/MS procedure, these components must be removed from the samples without appreciable protein loss.Recently we developed a facile, efficient, and reproducible precipitation/on-pellet-digestion procedure, which removes detergents, protease inhibitors, and non-protein matrix components efficiently by organic solvent precipitation without significant protein loss; then a 2-step enzymatic digestion procedure subsequently brings the precipitated proteins back into solution as soluble, completely-cleaved peptides, without introducing detergents [29].

The acetone precipitation procedure (e.g. the amount of acetone addition of the two-step precipitation), as well as on-pellet-digestion parameters (e.g. the enzyme-to-substrate ratio and the incubation durations), were optimized by monitoring the total ion currents and the completeness of digestion and of tryptic peptides generated in both digestion steps by nano-LC/LTQ/ETD and nano-LC/LTQ/Orbitrap. The optimized conditions were described in the Methods section. Under the optimized condition, the peptide recoveries from a bacterial lysate ranged from 87-93%, as determined by a revised BCA method we developed previously [29] (data not shown). This high and reproducible peptide recovery ensures a reliable proteomic comparison for bacteria grown in different conditions.

### Nano-LC/MS optimization

**Figure 1:**
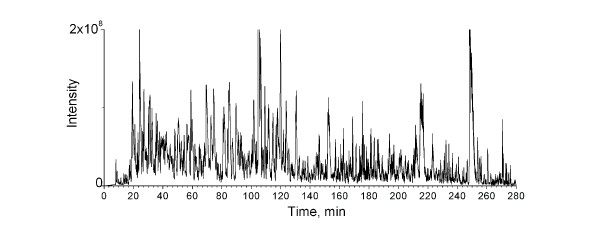
**Chromatogram showing elution gradient for the separation of bacterial lysate by Nano-flow liquid chromatography**. X- axis:elution time.Y-axis: Mass spectrometry signal intensity.

Because the whole bacterial lysate is highly complex, a large number of tryptic peptides are retrieved by the precipitation/on-pellet digestion procedure.As a result, sufficient chromatographic separation is required to achieve the most comprehensive identification/quantification of the proteome, especially for lower abundance peptides. To address this requirement, a chromatographic system with low void volume and high separation efficiency were employed with a shallow, long gradient (5 hour total separation time). A nano-LC, rather than a conventional LC, was used for peptide separation because of the significantly higher sensitivity, as we demonstrated previously [32,33]. As the high run-to-run reproducibility of retention times and MS signal intensities is essential [18], we employed a low-void-volume and high-resolution nano-LC/nanospray configuration with a non-coated fused silica tip (ID of 3 μm and an OD of 360 μm) that provides exceptional reproducibility [29].To achieve a comprehensive proteomic coverage, we used a relatively long (40 cm) reversed-phase nano-column in conjunction with a 5 hour, shallow elution gradient for the separation of bacterial lysate. A typical chromatogram is shown in Figure 1. An extended peptide elution window of more than 220 min was achieved, and this high level of chromatographic separation enabled extensive identification and profiling of the proteome.

### Evaluation of the quantitative methods

To investigate the performance of the method for a relatively large-scale expression profiling of bacterial lysate, the chromatographic reproducibility was evaluated with 12 repeated injections of the same pooled sample over a 3-day period. The reproducibility of chromatographic separation and signal intensities for the twelve 5-h runs was excellent, as assessed from data for selected tryptic peptides identified in the bacterial lysate preparation. Variations in retention time for the selected peptides were in the range of 0.32-1.05%, and variations for precursor ion current AUCs were in the range of 5-14% over the 3 day period. This high level of reproducibility can be attributed to two factors: (i) the highly reproducible chromatographic configuration described above, and (ii) the efficient precipitation/on-pellet-digestion procedure that removed detergents and other potentially interfering compounds.

Current methods for proteomic investigation are prone to false-positives arising from technical variability [34].In this study, to eliminate false-positives resulting from drift in nano-LC or ionization efficiency, for example, and possible instability of certain tryptic peptides, all samples were analyzed in a random order.To evaluate the false-positive rate before comparing the bacterial samples grown under different conditions, we designed an experiment to determine the false-positive rate in relative quantification. From the 10 repetitive analyses of a pooled bacterial sample (above), 5 runs were randomly assigned as the control group, and the remaining 5 were designated as the experimental group. Expression profiles between the two groups were then compared. In total, 32,178 ion-current frames were matched among the two groups of samples using Sieve. The observed distribution of peptide ratios (experimental:control) concentrated narrowly around 1.0, with 96% of ion-current frames in the range of 0.9-1.1. Approx. 1% of ions differed by more than 15% of the 1.0. Only 2 peptides were identified as significantly changed between the two groups at p < 0.05.Such a low false-positive rate and high quantitative precision supported the suitability of this method for profiling of the bacterial samples using the replicate number (n = 5) selected.

### Proteomic profiling of *H. influenzae *grown in chemically defined media with and without sputum

Previous analyses of the *H. influenzae *proteome have employed electrophoresis-based studies [35-40] to identify abundantly expressed proteins under laboratory growth conditions.More recently Kolker et al [41] employed a direct proteomics approach using liquid chromatography with ion trap tandem mass spectroscopy and identified 414 protein with high confidence, including 15 proteins that were encoded by genes that were previously annotated as conserved hypothetical proteins.

In the present study, we identified 1402 unique proteins with high confidence after application of a set of strict criteria, which included a stringent threshold for Xcorr, 10 ppm for precursor m/z tolerance, a peptide probability > 95%, a protein probability > 99%, and the requirement that two unique peptides must be identified for each protein (See Methods).These included 170 proteins that are encoded by genes that are annotated as conserved hypothetical and thus represent newly identified proteins in the proteome of *H. influenzae*.Analysis of the genome sequence of strain 11P6H predicts that the genome contains 1759 open reading frames, indicating that 79.6% of possible proteins were identified (Additional File 1).

Several methodological innovations likely account for the successful identification of 1402 proteins.The precipitation/on-pellet digestion followed by solubilization of peptide fragments is an efficient and reproducible method facilitating the recovery of proteins of varying solubilities from a complex mixture of proteins.The chromatographic system employed a low void volume and high separation efficiency with a shallow, long gradient (5 hour total separation time).Finally, a nano-LC for peptide separation allowed significantly higher sensitivity compared to conventional LC.This high level of proteomic coverage renders a comprehensive proteomic quantification.

### Ribosomal Proteins

Ribosomal proteins are among the most abundantly expressed protein types by cells.Therefore, the number of ribosomal protein identified allows an assessment of the proteomics methods.In the present study 47 of the known 54 ribosomal proteins of *H. influenzae *(87%) were detected with high confidence in cells that were grown in sputum (Additional File 2).Langen et al [38] employed two dimensional gel electrophoresis followed by identification with matrix-assisted laser desorption inonization-time of flight mass spectroscopy and detected 18 ribosomal proteins in *H. influenzae*.Kolker et al [41] identified 43 ribosomal proteins using liquid chromatography coupled with ion trap tandem mass spectrometry.In our study, all 7 of the ribosomal proteins that eluded detection were 100 amino acids or less in length and had isoelectric points of 10.1 or higher.We speculate the small size and/or the solubility characteristics of the proteins may have contributed to these proteins not being detected

### Proteins of the glycolysis pathway

Raghunathan et al [42] used an integrated approach to study intermediary metabolism of *H. influenzae *grown under microaerophilic and anaerobic conditions.Their analysis suggested that *H. influenzae *cells used glycolysis as the primary pathway of sugar metabolism during both growth conditions. In the present study, all proteins in the glycolysis pathway were detected with high confidence, suggesting that *H. influenzae *uses glycolysis during colonization of the human respiratory tract (Table 1).While growing bacteria in pooled human sputum simulates some conditions in the human respiratory tract and is an improvement over studying cells grown in laboratory media, one must be cautious in extrapolating results from cells grown in sputum to in vivo conditions.When *H. influenzae *inhabits the human respiratory tract, the organism is present in multiple locations, including embedded in mucous in the lumen, adhering to respiratory epithelial cells, inside epithelial cells and macrophages, and in the interstitium between cells.Thus, *H. influenzae *must adapt to the growth and metabolic conditions in multiple microenvironments and bacterial cells may express different proteins, depending on the microenvironment.Proteomic profiling of sputum-grown cells may represent an average of multiple conditions.

**Table 1:** Proteins of the glycolysis pathway identified in *H. influenzae *strain 11P6H identified during growth in pooled human sputum

Protein ID#	Identified Protein	Gene	**Genome ID number**^**a**^	Molecular Weight	**CDM**^**b**^	**Sputum**^**c**^
1237	phosphoenolpyruvate-protein phosphotransferase	*fruA*	HI0446	64 kDa	100% (9.7%)	100% (11%)

412	Fructose specific phosphotransferase system IIA/HPr components	*fruB*	HI0448	53 kDa	100% (24%)	100% (8.2%)

1149	Aldose 1-epimerase	*galM*	HI0818	38 kDa	100% (11%)	100% (15%)

423	1-phosphofructokinase	*fruK*	HI0447	34 kDa	100% (24%)	100% (15%)

557	6-phosphofructokinase	*pfkA*	HI0982	23 kDa	100% (21%)	95% (20%)

57	Fructose-bisphosphate aldolase	*fba*	HI0524	39 kDa	100% (47%)	100% (36%)

657	glucose-6-phosphate isomerase	*pgi*	HI1576	37 kDa	100% (19%)	100% (15%)

392	Triosephosphate isomerase	*tplA*	HI0678	27 kDa	100% (25%)	100% (19%)

97	Glyceraldehyde-3-phosphate dehydrogenase	*gapA*	HI0001	36 kDa	100% (40%)	100% (39%)

111	3-phosphoglycerate kinase	*pgk*	HI0525	41 kDa	100% (39%)	100% (37%)

34	phosphoglyceromutase	*gpmA*	HI0757	26 kDa	100% (52%)	100% (56%)

79	enolase	*eno*	HI0932	46 kDa	100% (43%)	100% (32%)

133	Pyruvate kinase	*pykA*	HI1573	49 kDa	100% (37%)	100% (47%)

538	Dihydrolipoamide acetyltransferase	*aceF*	HI1232	57 kDa	100% (22%)	100% (23%)

305	Pyruvate dehydrogenase subunit E1	*aceE*	HI1233	99 kDa	100% (28%)	100% (30%)

^a^ID numbers based on annotation of *H. influenzae *strain KW20 Rd http://cmr.jcvi.org/cgi-bin/CMR/GenomePage.cgi?org=ghi

^b^Protein probabilities values as calculated by Proteinprophet algorithm for proteins detected during growth in chemically define media (CDM). Number in parentheses represents the sequence coverage expressed by the percentage of amino acid residues identified. All peptides were filtered with a set of criteria as specified in the Methods. CDM

^c^Protein probabilities for proteins detected during growth in 20% pooled human sputum.

### Proteins expressed in increased amount during growth in sputum

**Figure 2:**
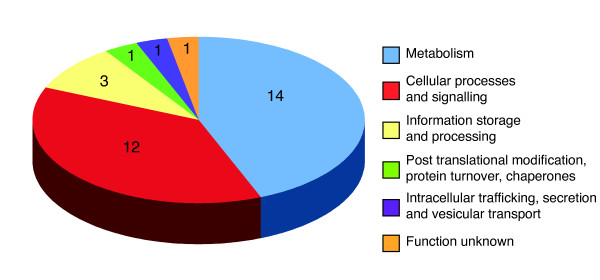
**Distribution of functional categories of 31 proteins that were present in increased abundance during growth of *H. influenzae *in 20% pooled human sputum compared to growth in chemically defined media**. Distribution of functional categories of 31 proteins that were present in increased abundance during growth of *H. influenzae *in 20% pooled human sputum compared to growth in chemically defined media. One protein is classified in two categories accounting for the total of 32.

Additional File 3 lists the 31 proteins that were present in a ratio of > 1.5 in sputum-grown compared to media-grown bacteria, along with the corresponding gene and the COG functional category.A range of proteins is represented but clear-cut themes are observed and these are shown graphically in Figure 2 and are outlined below.

In evaluating the proteins that are more abundant during growth in pooled human sputum supernatants, it is worth noting some limitations of this approach when interpreting the results.Because extracts were prepared from bacteria that were grown in liquid culture overnight, the differences in quantity reflect those in stationary phase cells.Logarithmic phase cells may differ in the proteins that are up regulated in expression compared to stationary phase cells.Bacterial populations that colonize the human respiratory tract are likely a mixture of bacteria in all phases of growth.

*H. influenzae *has been demonstrated to grow in the form of biofilms under in vitro conditions, in the middle ears of chinchillas and humans, and in the airways of children with cystic fibrosis [43-47].These observations indicate that biofilms play an important role in the pathogenesis of *H. influenzae *infections.Although *H. influenzae *biofilms have not yet been demonstrated directly in the airways of adults with COPD, many authors suggest that biofilms are present in this ecological niche and account, in part, for the recalcitrant nature of *H. influenzae *infections in COPD.Indeed, *H. influenzae *is likely present in the human airways in both planktonic and biofilm forms. It should be noted that the growth conditions used in the present study apply to planktonic bacteria, as cells were grown in liquid media.

Another limitation is that the sputum samples were homogenized in the reducing agent dithiotreitol before centrifugation and pooling.Taking into account the dilutions that were used to homogenize sputum and prepare media with 20% pooled sputum supernatant, the final concentration of dithiotreitol in the CDM plus sputum is 0.01%.It is interesting that several antioxidant proteins were present in increased abundance in the sputum grown cells in spite of the presence of the reducing agent in the culture (See below).We speculate that the small amount of reducing agent in the growth media was outweighed by the highly oxidative environment that is known to be present in human airways in COPD as reflected in pooled sputum from adults with COPD.

### Antioxidant proteins

Eight of the 31 proteins have stress or antioxidant functions, consistent with the observation that the airways in adults with COPD are an environment that induces an anti oxidant and stress response in *H. influenzae*.Three of these upregulated proteins encoded by *pdgX*, *trxA *and HI1349, have primary antioxidant functions.Of particular interest is peroxiredoxin-thioredoxin (*pdgX*) whose expression has previously been shown to be upregulated during biofilm formation by *H. influenzae *[48].Furthermore, adults with COPD who experience respiratory tract infection by *H. influenzae *develop new antibody responses to the peroxiredoxin-thioredoxin indicating that the protein is indeed expressed during infection and stimulates a human antibody response [48].The observation that the ratio of this protein in sputum-grown to media-grown *H. influenzae *(4.764) was among the highest detected in the present study is consistent with the observation that the protein is prominently expressed during infection and suggests that antioxidant activity is important for survival of *H. influenzae *in the airways.

### Stress response

Five stress related proteins were present in greater abundance during growth in sputum.These include GroEL, GroES, heat shock protein encoded by *dnaJ*, universal stress protein E and DNA-binding ferritin-like protein.The latter protein contains a DPS (**D**NA **p**rotein under **s**tarved conditions) domain which non specifically binds DNA, protecting it from cleavage by reactive oxygen species. The abundance of these proteins suggests that *H. influenzae *expresses a stress response during growth in the human respiratory tract.

### Uptake of nutrients and cofactors

In addition to the anti oxidant and stress response observed, several proteins that were present in greater abundance during growth in sputum function in uptake in nutrients and cofactors.Four such proteins function directly in uptake of divalent cations, including 3 iron uptake proteins (*yfeA, hitA, hxuB*) and one zinc uptake protein (*znuA*).The environment in the human host has exceedingly low concentrations of free iron; thus human pathogens have evolved mechanisms to scavenge iron during infection.These results indicate that *H. influenzae *grows in an iron stressed condition in the human respiratory tract.The presence of increased levels of several other proteins that function in transport of various nutrients and other molecules (proteins encoded by (*acpC, oppB, hslVU, uspE, pstB, tolQ, metQ, orfG*) indicates that the human respiratory tract is relatively deficient in nutrients causing *H. influenzae *to upregulate certain transport systems.

Gawronski et al [49] developed a novel approach of negative selection technology involving challenging mice with a mutant library of *H. influenzae *and identifying genes that were required to delay clearance of bacteria from the lungs.Genes that were implicated in survival in mouse lung included those that play potential roles in survival in nutrient limitation, oxidative stress and exposure to antimicrobial perturbations.While substantial differences between individual genes identified as important in mouse lungs compared to the proteins that were present in increased abundance in human sputum in the present study, the overall classes of genes/proteins show strong parallels.In particular, the expression in both systems of genes/proteins that function in survival in oxidative stress and nutrient limitation are consistent with the concept that these conditions exist in the respiratory tract and *H. influenzae *must express molecules to survive in these conditions in order cause respiratory tract infection.

### Urease

The gene that encodes the alpha subunit of urease, *ureC*, was present in 7 fold greater abundance in sputum grown conditions compared to media alone (Additional File 3).This is an interesting finding in light of the study by Mason et al [50] who monitored gene expression by nontypeable *H. influenzae *in the middle ear of chinchillas.The gene that encodes urease accessory protein, *ureH*, was induced 3.9 fold in bacterial cells in the middle ear compared to baseline.These two genes, *ureC *and *ureH *are part of the urease operon (*ureA, ureB, ureC, ureE, ureF, ureG, ureH*) and were among the most highly up regulated in the two studies involving two different conditions simulating human infection- the chinchilla middle ear and pooled human sputum.Urease catalyzes the hydrolysis of urea to produce CO_2 _and ammonia.The enzyme plays a role in acid tolerance and is a virulence factor in other bacteria including *Helicobacter pylori*, *Actinobacillus pleuropneumoniae, Yersinia enterocolitica *and *Morganella morganii *[51-55].We speculate that ureasemay function as a virulence factor for nontypeable *H. influenzae *by facilitating survival and growth in the relatively acid environment of the airways and middle ear.

### Adherence

The HMW1A protein is one of the major adhesins of *H. influenzae*, mediating adherence to respiratory epithelial cells [56,57].Indeed, HMW1 is one of the surface proteins that is a prominent target of human antibodies following infection caused by *H. influenzae *[58,59].The HMW1A adhesin was upregulated in sputum along with HMW1B which is an OMP85-like protein that functions specifically to facilitate secretion of the HMW1A adhesin.This result is consistent with the concept that adherence to respiratory epithelial cellsis critical in order for *H. influenzae *to colonize and infect the airways.

### Phosphoryl choline and lipooligosaccharide

Lipooligosaccharide is an abundant surface antigen that is involved in adherence, persistence and pathogenesis of *H. influenzae *infection.The *licD *gene encodes the enzyme phosphoryl transferease that adds phosphoryl choline to the lipooligosaccharide molecule.The *licD *gene product was upregulated 4.736 fold in sputum-grown compared to media grown bacteria (Additional File 3).This gene is part of the lic-1 protein operon (*licA, licB, licC, licD*) involved in lipooligosaccharide synthesis.In the study of gene expression by Mason et al [50], *licC *was 2.3 fold induced in the chinchilla middle ear.Herbert et al [60] identified *licC *as an essential gene in survival of *H. influenzae *type b in a model of systemic infection using signature tagged mutagenesis.The observation that the *lic *operon was identified in 3 independent model systems (pooled human sputum, chinchilla middle ear, infant rat) suggests that the lipooligosaccharide molecule, in particular addition of phosphoryl choline to lipooligosaccharide is important in pathogenesis. The present study extends this association into a human system for the first time, an important consideration in view of the fact that *H. influenzae *is an exclusively human pathogen.

Phosphoryl choline may participate in pathogenesis in several ways.Phosphoryl choline decreases the susceptibility of *H. influenzae *to antimicrobial peptides [61].Hong et al [62,63] demonstrated that phosphoryl choline promotes infection and persistence in an animal model by reducing the host inflammatory response and by promoting the formation and maturation of stable biofilm communities.Several indirect lines of evidence suggest that *H. influenzae *persists in the airway by forming biofilms that resist host immunity.The observation that the *licD *gene product is abundantly expressed in sputum suggests that addition of phosphoryl choline to lipooligosaccharide is important for persistence, perhaps by protecting the bacterial cell from antimicrobial peptides and/or by promoting the formation of biofilms.

## Conclusions

Proteomic expression profiling of a prototype COPD strain of *H. influenzae *was performed on bacteria that were grown in pooled human sputum in comparison to the same strain grown in defined chemical media.The sequence of the genome of the prototype strain was determined by pyrosequencing yielding 53 contigs.A method involving precipitation and on-pellet digestion of a whole bacterial cell lysate was optimized to solubilize proteins of varying solubilities from a complex mixture of proteins.

Proteomic profiling was accomplished using a Nano-LC/MS system and 1402 proteins were identified with high confidence using a set of strict criteria.These proteins represent 79.7% of the ORFs predicted from the genome sequence, including 170 proteins that are encoded by genes that are annotated as conserved hypothetical proteins.A total of 31 proteins were present in a ratio of > 1.5 in sputum grown compared to media grown bacteria.Analysis of these proteins reveal 8 antioxidant proteins and 5 stress response proteins, suggesting that expression of antioxidant activity and stress responses is important for survival of *H. influenzae *in the human airways.In addition, proteins involved in uptake of nutrients and adherence highlight the role of these possible functions for *H. influenzae *to survive in the human respiratory tract.

The results of proteomic expression profiling of *H. influenzae *grown in pooled human sputum from adults with COPD are revealing in understanding the adaptations that *H. influenzae *makes during colonization and infection of the human respiratory tract.These observations have the potential to reveal critical virulence factors that enable survival of *H. influenzae *in its ecological niche and may present opportunities for the development of novel approaches to interrupt infection.

## Methods

### Bacterial strain

Nontypeable *H. influenzae *strain 11P6H is a prototype exacerbation strain that was isolated from the sputum of an adult with chronic obstructive pulmonary disease (COPD).The strain was isolated simultaneous with the onset of clinical symptoms of an exacerbation and the patient subsequently developed a new serum bactericidal antibody response to the strain [64,65].

### Sputum supernatants

Expectorated sputum samples were collected from adults with COPD as part of other studies.All identifying information on samples was removed.Samples were processed for culture as previously described [66,67].Briefly, sputum samples from adults with COPD that had been spontaneously expectorated in the morning were homogenized by incubation at 37°C for 15 minutes with an equal volume of 0.1% dithiothreitol.After an aliquot was removed for quantitative culture, sputum supernatants were saved by centrifugation at 27,000 × g for 30 minutes at 4°C.Supernatants were stored at -80°C until used.Samples from patients who were receiving antibiotics and samples that grew potential pulmonary bacterial pathogens in culture were excluded.Supernatants from approximately 100 sputum samples from 30 individuals were pooled for the purpose of growing bacteria in pooled sputum supernatants.

To render the sputum supernatants sterile, the pooled samples were placed in Petri dishes and exposed to UV light in a cell culture hood for approximately 10 minutes.An aliquot was plated on chocolate agar and no growth was detected after overnight incubation.

### Growth conditions

*H. influenzae *strain 11P6H was grown overnight in 100 ml of chemically defined media (Table 2) at 37°C with shaking.A second 100 ml culture was grown simultaneously in CDM to which pooled human sputum supernatant of 20% of the volume of the culture was added.Cells were harvested by centrifugation at 10,000 × g for 10 minutes at 4°C.Cells were washed by suspending in cold phosphate buffered saline and centrifuging again using the same conditions.

**Table 2:** Composition of chemically defined media (CDM)

Reagent	Concentration
NaCl	0.1 M
K_2_SO_4_	5.75 mM
Na_2_EDTA	4 mM
NH_4_Cl	4 mM
K2HPO_4_	2 mM
KH_2_PO_4_	2 mM
Thiamine HCl	6 μM
Thiamine pyrophosphate	1 μM
Pantothenic acid	8 μM
d-Biotin	12 μM
Glucose	0.5%
Hypoxanthine	0.375 mM
Uracil	0.45 mM
L-aspartic acid	3.75 mM
L-glutamic acid HCl	7.5 mM
L-arginine	0.875 mM
Glycine HCl	0.225 mM
L-serine	0.475 mM
L-leucine	0.7 mM
L-isoleucine	0.225 mM
L-valine	0.525 mM
L-tyrosine	0.4 mM
L-cysteine HCl	0.35 mM
L-cystine	0.15 mM
L-proline	0.45 mM
L-tryptophan	0.4 mM
L-threonine	0.425 mM
L-phenylalanine	0.15 mM
L-asparagine	0.2 mM
L-glutamine	0.35 mM
L-histidine HCl	0.125 mM
L-methionine	0.1 mM
L-alanine	1.125 mM
L-lysine	0.35 mM
Glutathione reduced	0.15 mM
HEPES	42 mM
NaHCO_3_	0.125 mM
Na acetate trihydrate	6.25 mM
Choline chloride salt	0.05 mM
Myo-inositol	1 μM
MgCl_2_	2.5 mM
CaCl_2_	0.6 mM
Fe(NO_3_)_3_	0.1 mM
Nicotinamide adenine dinucleotide	0.02 mM
Protoporphyrin IX	0.02 mM
Histidine	6 μM
Triethanolamine	0.01%

### Whole bacterial cell preparation

Washed bacterial cells were suspended in 25 ml of extraction buffer (0.05 M tris-HCl, pH 8, 0.15 M NaCl, 2% nonidet P40, 0.5% sodium deoxycholate, 0.1% sodium dodecyl sulfate, 2 mM EDTA, 1 mM tris(2-carboxyethyl)phosphine hydrochloride [TCEP], Protease Inhibitor Cocktail Tablets [Roche] and Phosphatase Inhibitor Cocktail Tablets [Roche]).Cells were disrupted by twice passing them through a French pressure cell at 15,000 lb/in^2^.The suspension was centrifuged at 10,000 × g for 10 minutes at 4°C to remove unbroken cells. The supernatant was the whole bacterial cell preparation.The protein concentration was determined using the Microtiter Lowry Assay (Sigma).

### Whole genome sequencing

*H. influenzae *strain 11P6H was sequenced by 454-FLX pyrosequencing (Roche Applied Science, Indianapolis, IN) to 19-fold coverage across the genome.Sequence assembly was completed using 454 Newbler Assembler Software (Roche) and resulted in 53 contigs greater than 500 bp.Open reading frames were assigned with GeneMark.hmm http://opal.biology.gatech.edu/GeneMark/[68-70].The open reading frames were compared against the May 1, 2007 Genbank nr database using blastp [71].Significance was set at an e value of 1 x 10^-10 ^and the highest score for the blastp analysis was used for the initial protein annotation.

### Precipitation/on-pellet-digestion of bacterial cell preparation

To minimize false-positives, five aliquots each of the whole bacterial cell preparation of the CDM-grown and sputum-grown bacteria were prepared for each culture condition.Each sample was subjected individually to the gel-free, precipitation/on-pellet-digestion procedure developed previously [29].Briefly, extracts containing 150 μg of total protein in each sample (approximately 20 μl) were pipetted and transferred to a clean tube and then were precipitated by adding 40 μl of ice cold acetone (purity>99.99%, Puriss grade, Fluka).After vortexing, an additional 80 μl of acetone was added to each sample.Samples were vortexed and placed at -20°C overnight.

The samples were centrifuged at 10,000 × g for 15 minutes at 4°C.The acetone was removed and the pellets were air dried for 5 minutes.Pellets were suspended in 50 μl of50 mM tris, pH 8.5.A volume of 10 μl 0.25 mg/ml of activated TPCK-treated mass spectrometry grade trypsin (Trypsin Gold, Promega) was added.The samples were vortexed, centrifuged briefly to bring the sample to the bottom of the tube and incubated at 37°C with vortexing every hour.After 2 hours, another 10 μl of trypsin was added and the samples were incubated at 37°C for an additional ~5 hours with hourly vortexing.A volume of 3 μl of TCEP was added to each tube and incubated for 10 minutes at 37°C.A volume of 5 μl of freshly prepared iodoacetamide (Sigma) was added to samples and tubes were incubated for 30 minutes at 37°C in the dark.Samples were exposed to light for 15 minutes and then 25 μl of trypsin was added and samples were incubated overnight at 37°C.

### Nano-Liquid Chromatography/Mass Spectroscopy (Nano-LC/MS)

A nano-LC system consisting of a Spark Endurance autosampler (Emmen, Holland) and four Eksigent direct-flow capillary/nano-LC pumps (Dublin, CA) that were powered by pressurized nitrogen (110 p.s.i) were used for all analyses. In order to achieve a comprehensive separation of the complex peptide mixture, a nano-LC/nanospray setup, which features low void volume and high chromatographic reproducibility, was employed [29]. A reversed-phased peptide trap (300 μm I.D. x0.5 cm, Agilent, Palo Alto, CA) and a nano-LC column (50 μm I.D. × 40 cm, packed with Pepmap C18 sorbent) were used for peptide separation. The trap and the nano column were connected back-to-back on a Valco (Houston, TX) metal zero-dead-volume (ZDV) tee, and a waste line was connected to the 90° arm. Between the trap and the tee, a ZDV conductivity sensor (GE, Fairfield, CT) was connected to monitor the gradient change and trap washing efficiency. High voltage (1.7-2.5 kV) was applied to the metal tee for nanospray. Mobile phase A consisted of 0.1% formic acid in 2% acetonitrile and mobile phase B was 0.1% formic acid in 88% acetonitrile. The sample was loaded onto the trap with 3% B at a flow rate of 5 μL/min, and the trap was washed for 3 min. The valve was then switched to the analysis position, and the spray voltage was applied on the tee. A series of nano flow gradients was used; The flow rate was 200 nL/min and the gradient profile was (i) a linear increase from 3% to 9% B over 5 min; (ii) an increase from 9 to 23% B over 115 min; (iii) an increase from 23 to 35% B over 70 min; (iv) an increase from 35 to 60% B over 50 min; (v) an increase from 60 to 97% B in 35 min, and finally (vi) isocratic at 97% B for 25 min.

An LTQ/Orbitrap hybrid mass spectrometer (Thermo Fisher Scientific, San Jose, CA) was used for label-free quantification, and an LTQ/ETD (Thermo Fisher Scientific) was employed to evaluate the completeness of the digestion of the tryptic peptides. Both mass spectrometers were connected to the same nano-LC/Nanospray setup as described above. For LTQ/Orbitrap analysis, one scan cycle included an MS1 scan (m/z 300-2000) at a resolution of 60,000 followed by seven MS2 scans by LTQ, to fragment the seven most abundant precursors found in the MS1 spectrum. The target value for MS1 by Orbitrap was 3×10^6^. For LTQ/ETD, the MS was working under data-dependent mode; one scan cycle was comprised of an MS1 scan (m/z range from 300-2000) followed by six sequential dependent MS2 scans (the maximum injection time was 250 ms). The first, third, and fifth MS2 scans were CID fragmentations of the first, second, and third most-abundant precursors found in the MS1 spectrum, respectively. The second, fourth, and sixth MS2 scans were ETD fragmentations corresponding to the same group of precursors. For CID, the activation time was 30 ms, the isolation width was 1.5 amu, the normalized activation energy was 35%, and the activation *q *was 0.25. For ETD, a mixture of ultra-pure helium and nitrogen (25% helium and 75% nitrogen, purity > 99.995%) was used as the reaction gas. The ETD reaction time was set at 120 ms and the isolation width was 2 amu; supplemental activation, which uses a short CID activation process to dissociate the charge-stripped precursors, was employed to enhance the fragmentation efficiency for doubly-charged precursors. For both LTQ/ETD and LTQ/Orbitrap experiments, dynamic exclusion was used with one repeat count, 35s repeat duration, and 40s exclusion duration. All samples were analyzed in random order, in order to eliminate quantitative false-positives arising from peptide degradation and analytical artifacts such as possible drift in nano-LC or MS performance.

### Protein identification and quantification

Peptide/protein identification was first performed with BioWorks 3.3.1 embedded with Sequest (Thermo Scientific), against the genome sequence of *H. influenzae *strain 11P6H in the form of 53 contigs.The precursor mass tolerances were 10 ppm and 1.5 mass units, respectively, for Orbitrap and LTQ; the mass tolerance for the fragments of both CID and ETD was 1.0 unit. A stringent set of score filters was employed. Correlation score (Xcorr) criteria were as follows: ≥4 for quadruply-charged (4+) and higher charge states, ≥3 for 3+ ions, ≥2.2 for 2+ ions, and ≥1.7 for 1+ ions. The CID results were further analyzed using Scaffold 2 proteome software (Portland, OR) which integrates both Protein Prophet and Peptide Prophet: additional criteria were that two unique peptides must be identified independently for each protein, the peptide probability must be 95% or higher, and the protein probability must be 99% or higher.For ETD spectra, a final score (Sf) of 0.85 was required for each identification.

A commercial label-free quantification package, Sieve (Fiona build, v. 1.2, Thermofisher Scientific), was used for comparing relative abundance of peptides and proteins between the control and experimental groups. Briefly, the chromatographic peaks detected by Orbitrap were aligned and the peptide peaks were detected with a minimum signal intensity of 2×10^5^; peptide extracted ion current (XIC) peaks were matched by their retention time (± 1 min after peak alignment) and mass (± 0.025 unit) among sample runs. Each subset of matched peaks was termed a "frame".The area under the curve (AUC) of each matched peptide within a frame was calculated and compared to the corresponding peak in the control sample. Fisher's combined probability test was performed to determine whether there was any significant difference in peptide abundances between the two experimental groups. Relative abundance of an individual protein was calculated as the mean AUC ratio for all peptides derived from that protein. All proteins differing significantly between the two groups were confirmed by a stringent manual inspection of the fragmentation spectra and the XIC of the ions within a 3-min elution window.

## Abbreviations

AUC: area under curve; CDM: chemically define media; COG: cluster of orthologous groups; COPD: chronic obstructive pulmonary disease; LC: Liquid chromatography; MS: mass spectroscopy; psi: pounds per square inch; TCEP: tris(2-carboxyethyl)phosphine hydrochloride; UV: ultraviolet; Xcorr: correlation score; XIC: extracted ion current; ZDV: zero dead volume

## Authors' contributions

JQ had input into the design of the study, directed and performed the proteomics expression profiling, participated in data interpretation, wrote the proteomic sections of the manuscript, and contributed revisions to the final version of the manuscript. AJL had input into the design of the study, participated in data interpretation and contributed revisions to the final version of the manuscript. ALB had input into the design of the study, performed the proteomics expression profiling, participated in data interpretation and contributed revisions to the final version of the manuscript. JC performed the proteomics expression profiling, and participated in data interpretation. SRG performed the genome sequencing, participated in data interpretation and contributed revisions to the final version of the manuscript. TFM conceived the study, had input in the design, participated in data interpretation and wrote the manuscript. All authors read and approved the final manuscript.

## Supplementary Material

Additional file 1 **Proteins of *Haemophilus influenzae *strain 11P6H identified by proteomic expression profiling**. Column A. Protein number (arbitrary numbering) Column B. Highest score from BLAST search Column C. Molecular weight of protein Column D. Protein probabilities values as calculated by Proteinprophet algorithm for proteins detected during growth in chemically define media (CDM).Number in parentheses represents the sequence coverage expressed by the percentage of amino acid residues identified.All peptides were filtered with a set of criteria as specified in the Methods. Column E. Protein probabilities for proteins detected during growth in 20% pooled human sputum. Click here for file

Additional file 2 **Ribosomal proteins identified in *Haemophilus influenzae *strain 11P6H during growth in chemically defined media and pooled human sputum**. Column A. Protein number (arbitrary numbering) Column B. Ribosomal protein number Column C. Genome number.Numbers refer to *H. influenzae *strain KW20 Rd unless other wise noted. Column D. Molecular weight of protein Column E. Protein probabilities values as calculated by Proteinprophet algorithm for proteins detected during growth in chemically define media (CDM).Number in parentheses represents the sequence coverage expressed by the percentage of amino acid residues identified.All peptides were filtered with a set of criteria as specified in the Methods. Column E. Protein probabilities for proteins detected during growth in 20% pooled human sputum. Click here for file

Additional file 3 **Proteins expressed in greater abundance (> 1.5) during growth in sputum compared to media alone**. Column A. GenBank accession number of protein that yielded the highest score from a BLAST search.. Column B. Name of gene that encodes the protein. Column C. Ratio of protein quantity detected in sputum-grown to media-grown bacteria.. Column D. Function of protein. Column E. Cluster of orthologous group (COG). Column F. COG functional category. Click here for file
