# Supplementary material for: Proteomic expression profiling of Haemophilus influenzae grown in pooled human sputum from adults with chronic obstructive pulmonary disease reveal antioxidant and stress responses
Source: BMC Microbiol. 2010 Jun 1;10:162. doi: 10.1186/1471-2180-10-162 (PMC2887450; doi:10.1186/1471-2180-10-162)
Supplement: Additional file 2 — Ribosomal proteins identified in Haemophilus influenzae strain 11P6H during growth in chemically defined media and pooled human sputum. Column A. Protein number (arbitrary numbering) Column B. Ribosomal protein number Column C. Genome number.Numbers refer to H. influenzae strain KW20 Rd unless other wise noted. Column D. Molecular weight of protein Column E. Protein probabilities values as calculated by Proteinprophet algorithm for proteins detected during growth in chemically define media (CDM).Number in parentheses represents the sequence coverage expressed by the percentage of amino acid residues identified.All peptides were filtered with a set of criteria as specified in the Methods. Column E. Protein probabilities for proteins detected during growth in 20% pooled human sputum. [file 1471-2180-10-162-S2.DOC]

**Additional File 2.** Ribosomal proteins identified in *Haemophilus influenzae* strain 11P6H during growth in chemically defined media and pooled human sputum

| **ID #** | **Identified Proteins** | **Genome ID number^a^** | **Molecular Weight** | **CDM^b^** | **Sputum^c^** |
| --- | --- | --- | --- | --- | --- |
| 81 | 30S_ribosomal_protein_S1 | HI1220 | 60 kDa | 100% (42%) | 100% (33%) |
| 272 | 30S_ribosomal_protein_S2 | HI0913 | 26 kDa | 100% (29%) | 100% (43%) |
| 200 | 30S_ribosomal_protein_S3 | HI0783 | 26 kDa | 100% (32%) | 100% (24%) |
| 430 | 30S_ribosomal_protein_S4 | HI0801 | 23 kDa | 100% (24%) | 100% (24%) |
| 252 | 30S_ribosomal_protein_S5 | HI0795 | 18 kDa | 100% (30%) | 100% (38%) |
| 237 | 30S_ribosomal_protein_S6 | HI0547 | 15 kDa | 100% (30%) | 100% (44%) |
| 243 | 30S_ribosomal_protein_S7 | HI0580 | 18 kDa | 100% (30%) | 100% (48%) |
| 22 | 30S_ribosomal_protein_S8 | HI0792 | 14 kDa | 100% (57%) | 100% (42%) |
| 323 | 30S_ribosomal_protein_S9 | HI1442 | 15 kDa | 100% (27%) | 100% (21%) |
| 281 | 30S_ribosomal_protein_S10 | HI0776 | 14 kDa | 100% (29%) | 100% (46%) |
| 661 | 30S_ribosomal_protein_S11 | HI0800 | 14 kDa | 100% (19%) | 100% (24%) |
| 1195 | 30S_ribosomal_protein_S12 | HI0581 | 14 kDa | 99% (10%) | 100% (19%) |
| 33 | 30S_ribosomal_protein_S13 | HI0799 | 13 kDa | 100% (53%) | 100% (52%) |
| 454 | 30S_ribosomal_protein_S14 | HI0791 | 15 kDa | 100% (23%) | 100% (34%) |
| 1370 | 30S_ribosomal_protein_S15 | HI1328  HI1468 | 29 kDa | 100% (6.2%) | 100% (9.5%) |
| 27 | 30S_ribosomal_protein_S16 | HI0204 | 9 kDa | 100% (55%) | 100% (55%) |
| 691 | 30S_ribosomal_protein_S17 | HI0786 | 10 kDa | 100% (19%) | 100% (19%) |
| 205 | 30S_ribosomal_protein_S18 | HI0545 | 9 kDa | 100% (32%) | 100% (16%) |
| 581 | 30S_ribosomal_protein_S19 | HI0781 | 10 kDa | 98% (21%) | 100% (36%) |
| 375 | 30S_ribosomal_protein_S20 | HI0965  HI1320 | 10 kDa | 100% (25%) | 100% (38%) |
| 288 | 30S_ribosomal_protein_S21 | HI0531 | 8 kDa | 100% (28%) | 100% (31%) |
| 135 | 50S_ribosomal_protein_L1 | HI0516 | 24 kDa | 100% (37%) | 100% (35%) |
| 497 | 50S_ribosomal_protein_L2 | HI0780 | 30 kDa | 100% (22%) | 100% (33%) |
| 380 | 50S_ribosomal_protein_L3 | HI0777 | 18 kDa | 100% (25%) | 100% (25%) |
| 490 | 50S_ribosomal_protein_L4 | HI0778 | 22 kDa | 100% (22%) | 100% (32%) |
| 174 | 50S_ribosomal_protein_L5 | HI0790 | 20 kDa | 100% (34%) | 100% (20%) |
| 94 | 50S_ribosomal_protein_L6 | HI0793 | 19 kDa | 100% (41%) | 100% (33%) |
| 11 | 50S_ribosomal_protein_L7/L12 | HI0641 | 12 kDa | 100% (62%) | 100% (74%) |
| 89 | 50S_ribosomal_protein_L9 | HI0544 | 16 kDa | 100% (41%) | 100% (41%) |
| 116 | 50S_ribosomal_protein_L10 | HI0640 | 18 kDa | 100% (39%) | 100% (39%) |
| 455 | 50S_ribosomal_protein_L11 | HI0517 | 15 kDa | 100% (23%) | 100% (11%) |
| 30 | 50S_ribosomal_protein_L13 | HI1443 | 16 kDa | 100% (54%) | 100% (61%) |
| 45 | 50S_ribosomal_protein_L14 | HI0788 | 14 kDa | 100% (49%) | 100% (35%) |
| 131 | 50S_ribosomal_protein_L15 | HI0797 | 15 kDa | 100% (38%) | 100% (42%) |
| 1160 | 50S_ribosomal_protein_L16 | HI0784 | 15 kDa | 100% (11%) | 100% (32%) |
| 70 | 50S_ribosomal_protein_L17 | HI0803 | 15 kDa | 100% (45%) | 100% (47%) |
| 645 | 50S_ribosomal_protein_L18 | HI0794 | 13 kDa | 97% (20%) | 100% (31%) |
| 355 | 50S_ribosomal_protein_L19 | HI0201 | 13 kDa | 100% (26%) | 100% (43%) |
| 832 | 50S_ribosomal_protein_L20 | HI1320 | 13 kDa | 100% (16%) | 100% (16%) |
| 345 | 50S_ribosomal_protein_L21 | HI0880 | 11 kDa | 100% (26%) | 100% (33%) |
| 125 | 50S_ribosomal_protein_L22 | HI0782 | 12 kDa | 100% (38%) | 100% (36%) |
| 53 | 50S_ribosomal_protein_L24 | HI0789 | 11 kDa | 100% (48%) | 100% (41%) |
| 41 | 50S_ribosomal_protein_L28 | HI0951 | 9 kDa | 100% (50%) | 100% (44%) |
|  | 50S_ribosomal_protein_L29 | HI0785 | 7 kDa | 100%  (8.9%) | 100%  (18%) |
| 62 | 50S_ribosomal_protein_L31 | HI0758 | 8 kDa | 100% (46%) | 100% (43%) |
| 1230 | 50S_ribosomal_protein_L34 | HI0998 | 103 kDa (5 kDa)^d^ | 100% (9.9%) | 100% (5.8%) |
|  | 50S_ribosomal_protein_L35 | YP_001290792.1^e^ | 11 kDa | ND | 100%  (7.2) |

^a^ID numbers based on annotation of *H. influenzae* strain KW20 Rd unless otherwise noted (http://cmr.jcvi.org/cgi-bin/CMR/GenomePage.cgi?org=ghi)

^b^Protein probabilities values as calculated by Proteinprophet algorithm for proteins detected during growth in chemically define media (CDM). Number in parentheses represents the sequence coverage expressed by the percentage of amino acid residues identified. All peptides were filtered with a set of criteria as specified in the Methods. CDM

^c^Protein probabilities for proteins detected during growth in 20% pooled human sputum.

^d^Highest hit in GenBank was 50S ribosomal protein L34 from *H. influenzae* strain PittII (accession ZP_01793794) with a calculated molecular weight of 102,759. Corresponding protein in strain KW20 Rd (HI0998) is ribosomal protein L34 (Calculated molecular weight 5098).

^e^Highest hit in GenBank was 50S ribosomal protein L35 from *H. influenzae* strain PittEE which is not present in *H. influenzae* strain KW20 Rd.
