# Supplementary material for: Proteomic expression profiling of Haemophilus influenzae grown in pooled human sputum from adults with chronic obstructive pulmonary disease reveal antioxidant and stress responses
Source: BMC Microbiol. 2010 Jun 1;10:162. doi: 10.1186/1471-2180-10-162 (PMC2887450; doi:10.1186/1471-2180-10-162)
Supplement: Additional file 3 — Proteins expressed in greater abundance (> 1.5) during growth in sputum compared to media alone. Column A. GenBank accession number of protein that yielded the highest score from a BLAST search.. Column B. Name of gene that encodes the protein. Column C. Ratio of protein quantity detected in sputum-grown to media-grown bacteria.. Column D. Function of protein. Column E. Cluster of orthologous group (COG). Column F. COG functional category. [file 1471-2180-10-162-S3.DOC]

**Additional File 3.** Proteins expressed in greater abundance (ratio >1.5) during growth in sputum compared to media alone.

| **Accession number^1^** | **Gene** | **Ratio** | **Function** | **COG^2^** | **COG**  **Functional Category** |
| --- | --- | --- | --- | --- | --- |
| NP_439212.1 | HI1053 | 8.716 | Hypothetical protein | 0599 | S: Function unknown |
| YP_248248.1 | *ureC* | 7.012 | Urease subunit alpha | 0804 | E: Metabolism |
| ZP_00156390.1 | *pdgX* | 4.764 | Peroxiredoxin-thioredoxin | 0678 | O: Cellular processes and signaling |
| ZP_01788663.1 | *licD* | 4.736 | Phosphorylcholine transferase  (lipooligosaccharide biosynthesis) | 3475 | M: Cellular processes and signaling |
| NP_438524.1 | *yfeA* | 3.932 | Iron chelated ABC transporter periplasmic-binding protein | 0803 | P: Metabolism |
| AAB32110.1 | *hfbpA*  *hitA* | 2.543 | Iron utilization periplasmic protein | 1840 | P. Metabolism |
| YP_247982.1 | *hxuB* | 2.469 | Heme/hemopexin-binding protein | 2831 | U: Cellular processing and signaling |
| NP_438324.1 | *acpC* | 2.408 | Acyl carrier protein | 0236 | I: Metabolism |
| ZP_01789033.1 | *dnaJ* | 2.308 | Heat shock protein, molecular chaperone | 0484 | O: Cellular processing and signaling |
| ZP_00154813.1 | *trxA* | 2.138 | Thiol-disulfide isomerase and thioredoxins | 0526 | C: Cellular processing and signaling  O: Post translational modification, protein turnover, chaperones |
| YP_001293037.1 | *oppB* | 2.078 | Oligopeptide permease ABC transporter membrane protein | 4166 | E: Metabolism |
| ZP_01786105.1 | *hslVU* | 2.075 | ATP dependent protease ATP binding subunit | 1220 | O: Metabolism |
| ZP_01788540.1 | *purN*  *uspE* | 2.029 | Phosphoribosylglycinamide formyltransferase, universal stress protein E | 5266 | P: Metabolism |
| ZP_01791078.1 | groEL | 1.972 | Chaperonin GroEL | 0459 | O: Cellular processes and signaling |
| CAI77661.1 | *hmw1A* | 1.938 | Adhesin | 3210 | U: Intracellular trafficking, secretion and vesicular transport |
| ZP_00156515.1 | *clpP* | 1.883 | Protease subunit of ATP-dependent Clp proteases | 0740 | O and U: Cellular processes and signaling |
| ZP_01793054.1 | NTHI 0322 | 1.833 | Uracil-DNA glycosylase | 1573 | L: Information storage and processing |
| YP_001290359.1 | *znuA* | 1.819 | High-affinity zinc transporter periplasmic component | 4531 | P: Metabolism |
| ZP_00157218.2 | *pstB* | 1.808 | ABC-type phosphate transport system, ATPase component | 1117 | P: Metabolism |
| ZP_00154579.2 | *hmw1B* | 1.773 | Hemolysin activation/secretion protein | 2831 | U: Cellular processes and signaling |
| NP_438546.1 | *tolQ* | 1.747 | Colicin transport protein | 0811 | U: Cellular processes and signaling |
| YP_001290792.1 | *rpmI* | 1.718 | 50S ribosomal protein L35 | 0776 | L: Information storage and processing |
| ZP_00156362.1 | *groES* | 1.715 | Co-chaperonin GroES (HSP10) | 0234 | O: Cellular processes and signaling |
| ZP_01789526.1 | *gdhA* | 1.682 | glutamate_dehydrogenase | 0334 | E: Cellular metabolism |
| NP_439500.1 | HI1349 | 1.658 | DNA binding ferritin-like protein  (oxidative damage protectant) | 0783 | P: Metabolism |
| ZP_00154658.1 | *metQ* | 1.622 | D-methionine-binding lipoprotein | 1464 | P: Metabolism |
| YP_248913.1 | *prsA* | 1.600 | Ribose-phosphate pyrophosphokinase | 0462 | F: Metabolism |
| ZP_00155236.2 | *secD* | 1.566 | Preprotein translocase subunit SecD | 0342 | U: Cellular processes and signaling |
| ZP_00155435.2 | *orfG* | 1.560 | Putative DNA uptake protein | 0694 | O: Cellular processes and signaling |
| YP_248415.1 | *cyaA* | 1.522 | Adenylate_cyclase | 3072 | F: Metabolism |
| NP_438373.1 | *rpsP* | 1.508 | 30S ribosomal_protein S16 | 0228 | J: Information storage and processing |

^1^Protein that yielded the highest score from a BLAST search. Note that in the many instances, multiple genes yielded identical scores.

^2^Cluster of orthologous groups
